# Facile synthesis of symmetrical bis(benzhydryl)ethers using *p*-toluenesulfonyl chloride under solvent-free conditions

**DOI:** 10.1186/2191-2858-3-1

**Published:** 2013-02-18

**Authors:** Goutam Brahmachari, Bubun Banerjee

**Affiliations:** 1 Laboratory of Natural Products and Organic Synthesis, Department of Chemistry, Visva-Bharati University, Santiniketan, West Bengal, 731 235, India

**Keywords:** Bis(benzhydryl)ethers, Benzhydrols, *p*-Toluenesulfonyl chloride, Solvent-free

## Abstract

**Background:**

The benzhydryl ether moiety is widely distributed in nature and constitutes a key structural motif in numerous molecules of significant biological potential and of prospective clinical uses. Solvent-free and cost-effective facile synthesis of symmetrical bis(benzhydryl)ethers is, thus, much desirable.

**Results:**

A simple and efficient method for the facile synthesis of symmetrical bis(benzhydryl)ethers directly from the corresponding benzhydrols has been developed using a catalytic amount of *p*-toluenesulfonyl chloride (5 mol%) at an oil bath temperature of 110°C under solvent-free conditions.

**Conclusions:**

Operational simplicity, low reagent loading, high product yields, short reaction time, and solvent-free conditions are the notable advantages of the present method.

## Background

The benzhydryl ether moiety is abundant in a number of naturally occurring and biologically active compounds as well as molecules of potential clinical uses [1-8]; this motif was also found as a partial structure in a few new chemical entities showing therapeutic activity as well [9]. A number of reports are available describing the synthesis of molecules bearing this structural motif, which were shown to exhibit various pharmacological potentials such as non-nucleoside reverse transcriptase inhibition [10], anti-plasmodial and anti-trypanosomal action [11], monoamine uptake inhibition, anti-depressant and anti-parkinsonian activity [12,13], and anti-histaminic [14] and anti-spasmodic [15] action. Naturally occurring symmetrical bis(benzhydryl)ethers are also known to show promising therapeutic potentials including significant anti-platelet aggregation efficacy [16]. Very recently, application of such ether substructures in the total syntheses of a number of natural products has nicely been reviewed by Pitsinos et al. [17]. Although there are a good number of reports on the synthetic methodology of diaryl ethers, there are only two such reports so far on bis(benzhydryl)ethers in the literature [18-20]; symmetrical bis(benzhydryl)ethers were conventionally synthesized from corresponding benzhydrols using 100% sulfuric acid in large excess [18-20] and *p*-toluenesulfonic acid in equivalent amount [21]. Both of these earlier methods require the use of strong acids in relatively large excess. Under this purview, we have been motivated to undertake systematic planning to develop a convenient and efficient protocol for the conversion of benzhydrols into their bis(benzhydryl)ether derivatives.

**Scheme 1:**
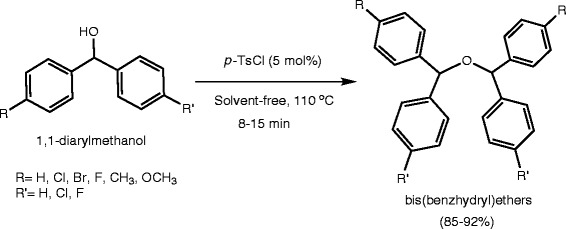
Synthesis of symmetrical bis(benzhydryl)ethers.

In continuation of our effort to develop green and solvent-free synthetic methodologies for organic transformations [22-28], we wish to report in this communication a convenient and straightforward protocol for the efficient synthesis of symmetrical bis(benzhydryl)ethers in excellent yields using a catalytic amount of *p*-toluenesulfonyl chloride under solvent-free conditions (Scheme 1). The process is very simple, cost-effective, and environmentally benign.

## Methods

Infrared spectra were recorded using a Shimadzu (FT-IR 8400S) Fourier transform infrared (FT-IR) spectrophotometer (Shimadzu, Kyoto, Japan) using KBr disc. ^1^H and ^13^C nuclear magnetic resonance (NMR) spectra were obtained at 400 and 100 MHz, respectively, using a Bruker DRX400 spectrometer (Bruker Instruments, Billerica, MA, USA) and CDCl_3_ as the solvent. Mass spectra (time-of-flight mass spectrometry (TOF-MS)) were measured on a Q-Tof Micro™ mass spectrometer (Waters MS Technologies, Manchester, UK). Elemental analyses were performed with an Elementar Vario EL III Carlo Erba 1108 micro-analyzer instrument (Carlo Erba Reagenti SpA, Rodano, Italy). Melting point was recorded on a Sunvic melting point apparatus (Sunvic, Glasgow, UK) and is uncorrected. Column chromatography was carried out over silica gel (60 to 120 mesh, Merck & Co., Inc., Whitehouse Station, NJ, USA), and thin layer chromatography (TLC) was performed using silica gel 60 F_254_ (Merck) plates.

## Results and discussion

**Figure 1:**
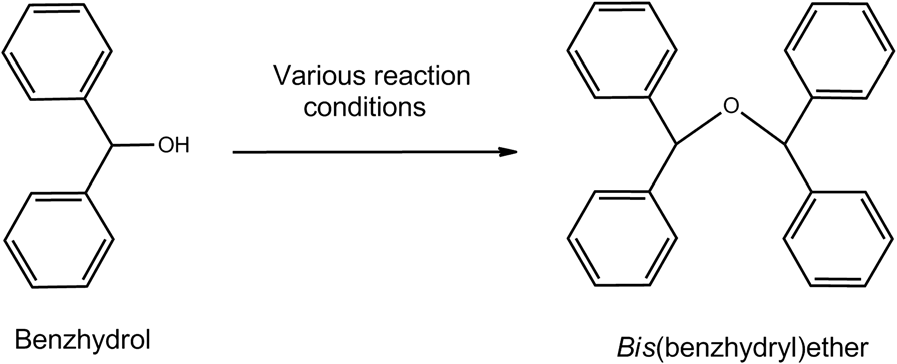
Optimization of the reaction conditions.

Firstly, we carried out the synthesis of bis(benzhydryl)ether **1** from benzhydrol as our model reaction in order to optimize the best suited reaction conditions (Figure 1); it was observed (Table 1) that the alcohol in the presence of *p*-TsCl (5 mol%) afforded the best result with 86% isolated yield at 110°C within a short period of time (15 min) under solvent-free conditions.

**Table 1:** **Optimization of the reaction conditions following Figure**1

**Entry**	***p*-TsCl (mol%)**	**Solvent**	**Temperature (°C)**	**Time (min)**	**Yield (%)**^**a**^
1	0	-	110	120	No reaction
2	10	-	90	25	82
3	10	-	110	15	87
4	5	-	90	35	80
*5*	*5*	-	*110*	*15*	*86*
6	5	-	Rt	240	Trace
7	3	-	110	75	28
8	10	CH_2_Cl_2_	Rt	1,050	19
9	5	CH_2_Cl_2_	Rt	1,050	Trace
10	10	THF	Rt	600	Trace
11	10	CH_3_CN	Rt	720	17
12	10	CH_2_Cl_2_	Reflux	300	36
13	50	-	110	5	43 (tosylate: 47)
14	100	-	110	5	7 (tosylate: 91)

^a^Isolated yield; RT, room temperature; *p*-TsCl, *p*-toluenesulfonyl chloride; Tosylate, benzhydryl *p*-toluenesulfonate.

**Figure 2:**
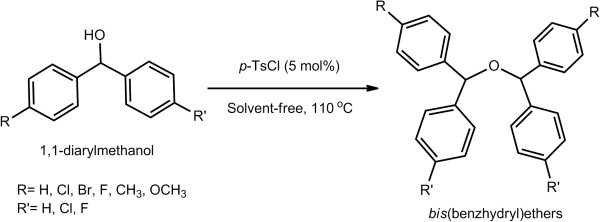
**Synthesis of symmetrical bis(benzhydryl)ethers using *p*-TsCl as reagent under solvent-free conditions.**

**Figure 3:**
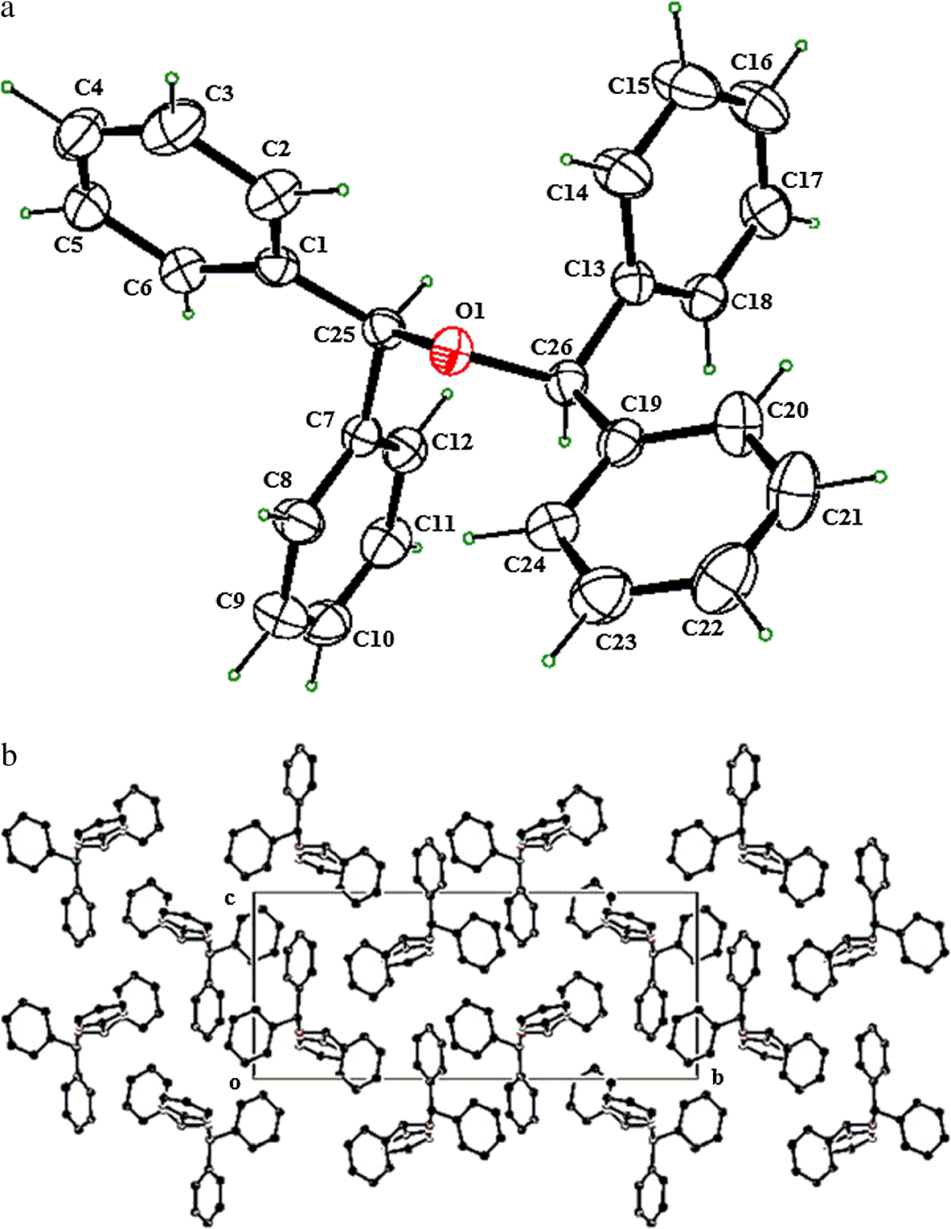
**Diagram and packing arrangement.** (**a**) ORTEP diagram of compound **1** (CCDC 840259). (**b**) The packing arrangement of molecules viewed down the a-axis.

A number of benzhydrol derivatives containing mono- and di-chloro, mono-bromo, di-fluoro, mono-methoxy, and mono-methyl phenyl groups were then screened for studying the generality as well as the efficacy of this present procedure (Figure 2; Table 2). All the entries find an easy and efficient route to their symmetrical bis(benzhydryl)ether derivatives in the presence of *p*-TsCl under solvent-free conditions (Figure 2) within 8 to 15 min affording excellent yields (85% to 92%). The workup of the reaction mixtures is simple and highly convenient. Each product has been characterized by detailed spectral analyses including FT-IR, ^1^H NMR, ^13^C NMR, and TOF-MS. In addition, the molecular structure of bis(bis*-*phenylmethyl)ether (Table 2, entry 1) has unambiguously been confirmed from X-ray crystallographic analysis [29-33] (Figure 3).^a^

**Table 2:** **Synthesis of symmetrical bis(benzhydryl)ethers using *p*-TsCl as reagent under solvent-free conditions following Figure**2

**Entry**	**Alcohol**	**Product**	**Time (min)**	**Yield (%)**^**a**^	**Melting point (°C)**	
					**Found**	**Reported**
1			15	86	106 to 107	105 to 107 [34]
2			15	89	Semisolid	Present work
3			10	85	Semisolid	Present work
4			10	92	Semisolid	Present work
5			10	88	125 to 127	126 to 127 [34,35]
6			8	91	88 to 90	Present work
7			12	90	Semisolid	Present work

^a^Isolated yield.

**Scheme 2:**
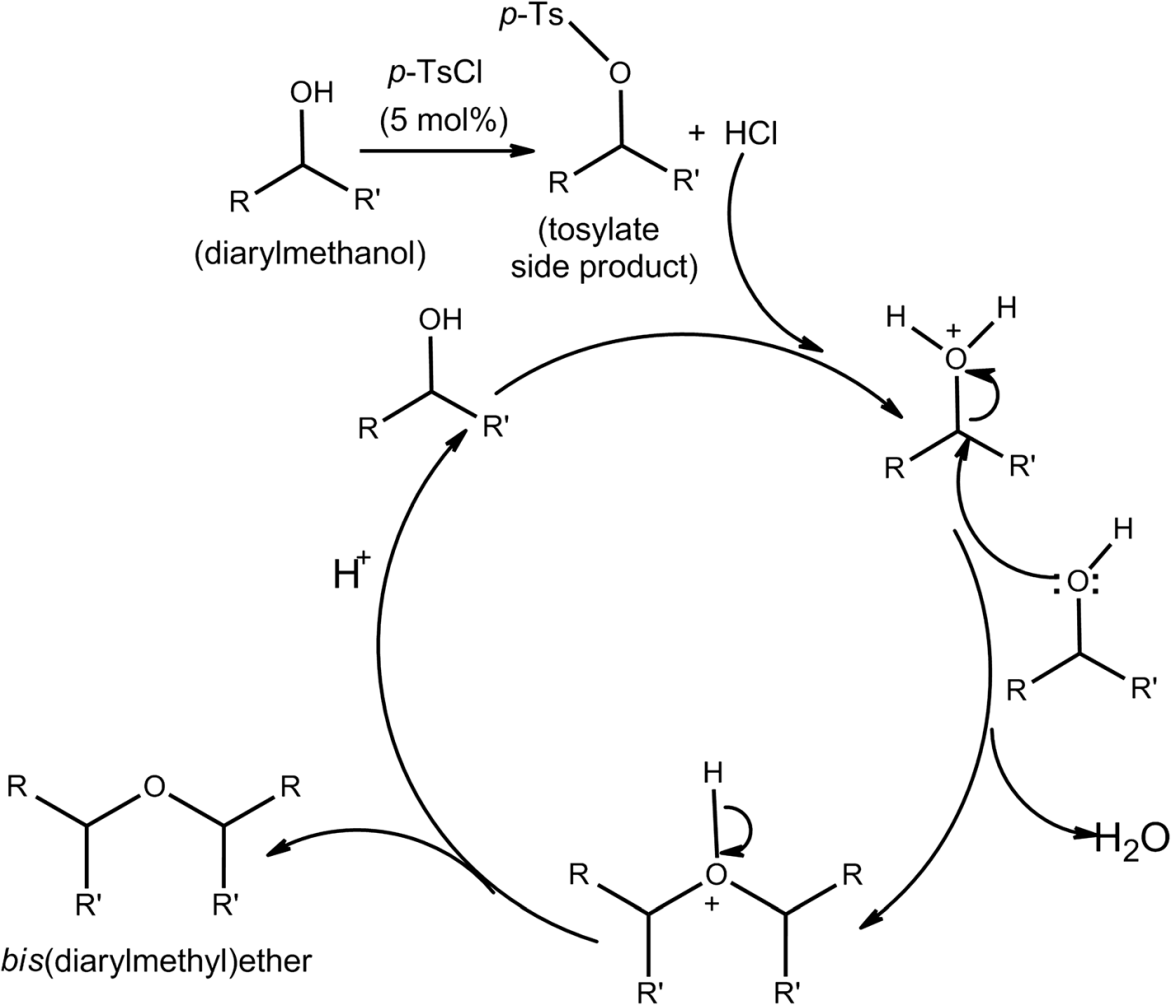
Proposed mechanistic pathway for etherification.

We propose the following mechanistic pathway for the reaction (Scheme 2). *p*-TsCl reacts rapidly with an equivalent amount of diarylmethanol to generate HCl (and a tosylate derivative as side product) *in situ* that eventually catalyzes the etherification following a catalytic cycle. The corresponding tosylate derivative remains intact as side product. To ensure the fact, we have checked the reaction with 50 and 100 mol% *p*-TsCl in two separate entries (entries 13 and 14; Table 1) where benzhydryl *p*-toluenesulfonate was isolated as 47% and 91% yields, respectively. In addition, we have carried out the reaction with benzhydrol separately using dry HCl gas (passed for a while into the reaction vessel) and concentrated hydrochloric acid; it has been found that dry HCl could also act as an efficient catalyst producing the corresponding ether derivative **1** with 82% yield in 30 min at 110°C, while concentrated HCl (45 mg added to 1 mmol of benzhydrol) required much more time (45 min) producing less amount of yield (62%) at the same reaction temperature. These experimental observations support our proposed mechanistic pathway as well.

## Experimental

### General procedure for the synthesis of symmetrical bis(benzhydryl)ethers (entries 1 to 7)

An oven-dried screw cap test tube was charged with a magnetic stir bar, benzhydrol (1 mmol), and *p*-toluenesulfonyl chloride (5 mol%). The tube was then evacuated and back-filled with nitrogen. The evacuation/backfill sequence was repeated two additional times. The tube was placed in a preheated oil bath at 110°C, and the reaction mixture was stirred vigorously. The progress of the reaction was monitored by TLC, and on completion, the reaction mixture was cooled to room temperature. The reaction mixture was extracted with dried ethyl acetate (10 ml), and the extract was then concentrated under reduced pressure; the residue was purified via column chromatography using silica gel (60 to 120 mesh) and petrol ether-ethyl acetate mixture. The structure of each purified symmetrical bis(benzhydryl)ethers was confirmed by analytical as well as spectral studies including FT-IR, ^1^H NMR, ^13^C NMR, and TOF-MS. Respective physical and spectral properties of bis(diarylmethyl)ethers are described below.

The spectral and analytical data of all the compounds including all new entries are given below (see also Additional file 1):

•*Bis(bis-phenylmethyl)ether (1)*: white solid, 86% yield, m.p. 106°C to 107°C (Lit. 105°C to 107°C [34]. 107°C [18]). IR (*ν*_max_, KBr) cm^-1^: 3,057, 3,028, 2,953, 1,595, 1,489, 1,445, 1,250, 1,163, 1,098, 1,072, 1,029, 6,98. ^1^H NMR (CDCl_3_, 200 MHz, δ): 7.40 to 7.23 (m, 20H, Ar H), 5.41 (s, 2H, CH). ^13^C NMR (CDCl_3_, 100 MHz, δ): 142.28, 128.45, 127.51, 127.33, 80.05. TOF-MS: 373.44 ([M + Na]^+^). Anal. found: C, 89.13; H, 6.28. C_26_H_22_O requires C, 89.11; H, 6.33%

•*Bis[[1-(4-methylphenyl)-1-phenyl]methyl]ether (2)*: yellowish white, semi solid, 89% yield. IR (*ν*_max_, KBr) cm^-1^: 3,060, 3,025, 2,923, 2,852, 1,655, 1,460, 1,277, 1,124, 1,071, 824, 810, 699. ^1^H NMR (CDCl_3_, 400 MHz, *δ*): 7.6 (d, 4H, Ar H, *J* = 7.6 Hz), 7.53 to 7.45 (m, 8H, Ar H), 7.43 to 7.41 (m, 2H, Ar H), 7.34 (d, 4H, Ar H, *J* = 7.6 Hz), 5.63 (s, 2H, CH), 2.53 (s, 6H, CH_3_). ^13^C NMR (CDCl_3_, 100 MHz, δ): 142.82, 142.70, 139.59, 139.48, 137.26, 137.22, 129.33, 129.30, 128.57, 128.54, 127.53, 127.49, 127.46, 127.41, 127.34, 79.96, 21.37. TOF-MS: 401.05 ([M + Na]^+^). Anal. found: C, 89.89; H, 6.90. C_28_H_26_O requires C, 89.85; H, 6.92%

•*Bis[[1-(4-chlorophenyl)-1-phenyl]methyl]ether (3)*: white semi solid, 85% yield. IR (*ν*_max_, KBr) cm^-1^: 3,063, 3,029, 2,925, 2,854, 1,595, 1,490, 1,449, 1,259, 1,185, 1,086, 1,057, 843, 811, 700. ^1^H NMR (CDCl_3_, 400 MHz, *δ*): 7.31 to 7.30 (m, 8H, Ar H), 7.28 to 7.25 (m, 10H, Ar H), 5.33 (s, 2H, CH). ^13^C NMR (CDCl_3_, 100 MHz, *δ*): 141.49, 141.39, 140.65, 140.54, 133.37, 133.30, 128.69, 128.63, 128.60, 128.56, 128.48, 127.87, 127.81, 127.22, 127.13, 79.53. TOF-MS: 441.94 ([M + Na]^+^). Anal. found: C, 74.45; H, 4.83. C_26_H_20_Cl_2_O requires C, 74.47; H, 4.81%

•*Bis[[1-(4-bromophenyl)-1-phenyl]methyl]ether (4)*: white semi solid, 92% yield. IR (*ν*_max_, KBr) cm^-1^: 3,085, 3,062, 3,028, 2,924, 2,854, 1,602, 1,590, 1,486, 1,454, 1,290, 1,185, 1,107, 1,070, 1,028, 847, 793, 700. ^1^H NMR (CDCl_3_, 400 MHz, *δ*): 7.33 (dd, 4H, Ar H, *J* = 8.4, 5.2 Hz), 7.21 to 7.15 (m, 10H, Ar H), 7.12 (dd, 4H, Ar H, *J* = 8.4, 3.2 Hz), 5.23 (s, 2H, CH). ^13^C NMR (CDCl_3_, 100 MHz, δ): 141.43, 141.33, 141.20, 141.08, 131.68, 131.62, 128.94, 128.86, 128.70, 128.65, 127.94, 127.87, 127.26, 127.17, 121.60, 121.52, 79.61. TOF-MS: 528.74 ([M + Na]^+^). Anal. found: C, 61.49; H, 3.93. C_26_H_18_Br_2_O requires C, 61.44; H, 3.97%

•*Bis[bis(4-chlorophenyl)methyl]ether (5)*: white solid, 88% yield, m.p. 125°C to 127°C (Lit. 126°C to 127°C) [35,36]. IR (*ν*_max_, KBr) cm^-1^: 3,031, 2,924, 1,594, 1,491, 1,410, 1,290, 1,188, 1,089, 1,013, 854, 824, 735, 726. ^1^H NMR (CDCl_3_, 200 MHz, *δ*): 7.31 (d, 8H, Ar H, *J* = 8.6 Hz), 7.23 (d, 8H, Ar H, *J* = 8.6 Hz), 5.29 (s, 2H, CH). ^13^C NMR (CDCl_3_, 75 MHz, *δ*): 139.72, 133.71, 128.82, 128.36, 78.97. TOF-MS: 509.12 ([M + Na]^+^). Anal. found: C, 63.94; H, 3.69; C_26_H_18_Cl_4_O requires C, 63.96; H, 3.72%

•*Bis[bis[4-fluorophenyl]methyl]ether (6)*: white solid, 91% yield, m.p. 88°C to 90°C. IR (*ν*_max_, KBr) cm^-1^: 3,069, 3,057, 2,925, 1,603, 1,507, 1,422, 1,408, 1,298, 1,225, 1,178, 1,155, 1,101, 1,029, 859, 837, 818. ^1^H NMR (CDCl_3_, 400 MHz, δ): 7.19 to 7.16 (m, 8H, Ar H), 6.94 to 6.88 (m, 8H, Ar H), 5.22 (s, 2H, CH). ^13^C NMR (CDCl_3_, 100 MHz, *δ*): 163.52, 161.07, 137.51, 137.48, 128.82, 128.74, 115.59, 115.38, 78.91. TOF-MS: 445.98 ([M + Na]^+^). Anal. found: C, 73.89; H, 4.28. C_26_H_18_F_4_O requires C, 73.93; H, 4.30%

•*Bis[[1-(4-methoxyphenyl)-1-phenyl]methyl]ether (7)*: colorless liquid, 90% yield. IR (*ν*_max_, KBr) cm^-1^: 3,062, 3,029, 2,953, 2,932, 2,906, 2,835, 1,510, 1,494, 1,451, 1,249, 1,171, 1,111, 1,080, 849, 819, 698. ^1^H NMR (CDCl_3_, 400 MHz, *δ*): 7.35 (d, 4H, Ar H, *J* = 7.6 Hz), 7.32 to 7.28 (m, 4H, Ar H), 7.27 to 7.24 (m, 6H, Ar H), 6.84 (d, 4H, Ar H, *J* = 8.4 Hz), 5.34 (s, 2H, CH), 3.77 (s, 6H, OCH_3_). ^13^C NMR (CDCl_3_, 100 MHz, *δ*): 158.97, 158.93, 142.72, 142.52, 134.53, 134.32, 128.67, 128.60, 128.35, 128.32, 127.30, 127.24, 127.17, 127.09, 113.80, 113.77, 79.43, 79.40, 55.26. TOF-MS: 432.99 ([M + Na]^+^). Anal. found: C, 81.95; H, 6.37. C_28_H_26_O_3_ requires C, 81.92; H, 6.38%

## Conclusions

In conclusion, we have developed a very simple and highly efficient solvent-free protocol for the synthesis of symmetrical bis(benzhydryl)ethers using inexpensive *p*-toluenesulfonyl chloride as reagent. The significant features of this environmentally benign and cost-effective straightforward protocol for direct conversion of benzhydrols into symmetrical bis(benzhydryl)ethers include operational simplicity, low reagent loading, high product yields, short reaction time, and solvent-free conditions.

## Endnote

^a^The molecular structure of the product, bis(bis*-*phenylmethyl)ether (**1**), was determined by means of X-ray crystallographic studies. CCDC 840259 (**1**) contains the supplementary crystallographic data for this article. These data can be obtained free of charge from The Cambridge Crystallographic Data Centre via http://www.ccdc.cam.ac.uk/data_request/cif.

## Competing interests

The authors declare that they have no competing interests.

## Supplementary Material

Additional file 1 ** Supplementary information Description: A document showing the general experimental details and procedures for the synthesis of symmetrical bis(benzhydryl)ethers.** Copies of ^1^H- and ^13^C-NMR spectra of all the entries (**1** to **7**) are also supplied. Click here for file
