# Supplementary material for: Facile synthesis of symmetrical bis(benzhydryl)ethers using p-toluenesulfonyl chloride under solvent-free conditions
Source: Org Med Chem Lett. 2013 Feb 18;3:1. doi: 10.1186/2191-2858-3-1 (PMC3637510; doi:10.1186/2191-2858-3-1)
Supplement: Additional file 1 — Supplementary information Description: A document showing the general experimental details and procedures for the synthesis of symmetrical bis(benzhydryl)ethers. Copies of 1H- and 13C-NMR spectra of all the entries (1 to 7) are also supplied. [file 2191-2858-3-1-S1.doc]

**Paper**

**Supplementary Information**

**Facile synthesis of symmetrical *bis*(benzhydryl)ethers using**

***p*-toluenesulfonyl chloride under solvent-free conditions**

Goutam Brahmachari^1^*****

*Corresponding author

E-mail: [brahmg2001@yahoo.co.in](mailto:brahmg2001@yahoo.co.in)

[brahmg2001@gmail.com](mailto:brahmg2001@gmail.com)

Bubun Banerjee^1^

E-mail: banerjeebubun@gmail.com

**In memory of Santosh Kr. Brahmachari**

^1^Laboratory of Natural Products & Organic Synthesis, Department of

Chemistry, Visva-Bharati University, Santiniketan-731 235, West Bengal,

India. Tel./Fax: 91-3463-261526

**General Experimental Details.** Infrared spectra were recorded using a Shimadzu (FT-IR 8400S) FT-IR spectrophotometer using KBr disc. ^1^H and ^13^C NMR spectra were obtained at 400 MHz and 100 MHz, respectively, using a Bruker DRX400 spectrometer and CDCl_3_ as the solvent. Mass spectra (TOF-MS) were measured on a Qtof MicroTM mass spectrometer. Elemental analyses were performed with an Elementar Vario EL III Carlo Erba 1108 micro-analyzer instrument. Melting point was recorded on a Sunvic melting point apparatus and is uncorrected. Column chromatography was carried out over silica gel (Merck 60-120 mesh) and TLC was performed using silica gel 60 F_254_ (Merck) plates. The spectral and analytical data of all the entries 1-7 are given.

**General procedure for the synthesis of symmetrical *bis*(benzhydryl)ethers in the presence of catalyst (compounds 1-7).** An oven-dried screw cap test tube was charged with a magnetic stir bar, benzhydrol (1 mmol) and *p*-toluenesulfonyl chloride (5 mol%). The tube was then evacuated and back-filled with nitrogen. The evacuation/backfill sequence was repeated two additional times. The tube was placed in a preheated oil bath at 110 ^o^C and the reaction mixture was stirred vigorously. The progress of the reaction was monitored by TLC, and on completion the reaction mixture was cooled to room temperature. The reaction mixture was extracted with dried ethyl acetate (10 ml), and the extract was then concentrated under reduced pressure; the residue was purified *via* column chromatography using silica gel (60-120 mesh) and petrol ether-ethyl acetate mixture. The structure of each purified symmetrical *bis*(benzhydryl)ethers was confirmed by analytical as well as spectral studies including FT-IR, ^1^H NMR, ^13^C NMR and TOFMS.

**X-ray study of *bis(bis-*phenylmethyl)ether** (**1**).

X-ray intensity data of were collected on *X’calibur*  CCD area-detector diffractometer equipped with graphite monochromated MoKα radiation (λ = 0.71073 Å). The crystal used for data collection was of dimensions 0.30 × 0.20 × 0.20 mm. The cell dimensions were determined by least-squares fit of angular settings of 11721 reflections in the θ  range 3.60 to 28.90^o^**.** The intensities were measured by ω scan mode for θ  ranges 3.61 to 25.00^o^. 2672 reflections were treated as observed (I > 2σ(I)). Data were corrected for Lorentz, polarisation and absorption factors. The structure was solved by direct methods using SHELXS97.^[1]^ An ORTEP view of the title compound with atomic labeling is shown in Fig.1.^[2]^. The geometry of the molecule was calculated using the WinGX ^[3]^ and PARST ^[4]^ and PLATON ^[5]^ softwares. All non-hydrogen atoms of the molecule were located in the best E-map. Full-matrix least-squares refinement was carried out using SHELXL97.^[15a]^ The final refinement cycles converged to an R = 0.0438 and wR (F^2^) = 0.1020 for the observed data. Residual electron densities ranged from −0.120 to 0.125 eÅ^−3^. Compound **1** (Table 2, entry 1) empirical formula C_26_H_22_O, white blocked shaped crystal, formula wt 350.44, space group P2_1_/n with the unit-cell parameters: a= 9.2922(3), b= 22.6718(7), c= 9.8383(3) Å, β = 104.905(3)^o^ and Z = 4. Complete crystallographic data of *bis(bis-*phenylmethyl)ether (**1**) (Table 2, entry 1) for the structural analysis have been deposited with the Cambridge Crystallographic Data Centre, CCDC No. 840259 . Copies of this information may be obtained free of charge from The Cambridge Crystallographic Data Centre *via* www.ccdc.cam.ac.uk/data_request/cif.

| 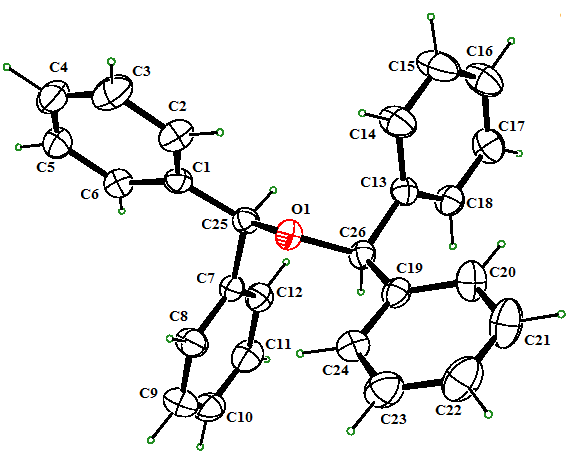  **Fig. 1a.** ORTEP diagram of compound **1**  (CCDC 840259) | **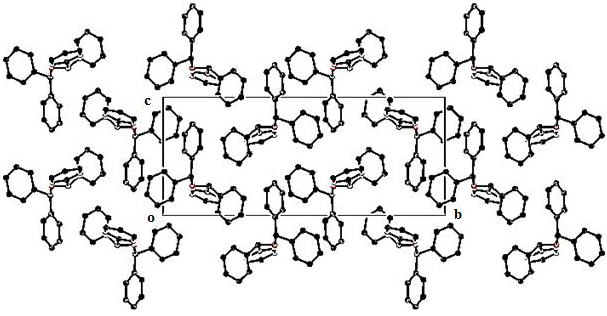**  **Fig. 1b.** The packing arrangement of  molecules viewed down the a-axis. |
| --- | --- |

**Table 1.** Crystal and experimental data for *bis(bis-*phenylmethyl)ether (**1**).

__________________________________________________________________

CCDC No 840259

Crystal description Block shaped

Crystal colour White

Crystal size 0.30 x 0.20 x 0.20 mm

Empirical formula C26 H22 O

Formula weight 350.44

Radiation, Wavelength Mo *K*α, 0.71073 Å

Unit cell dimensions a= 9.2922(3), b= 22.6718(7), c= 9.8383(3) Å,

β = 104.905(3)^o^

Crystal system Monoclinic

Space group P21/n

Unit cell volume 2002.91(11)

No. of molecules per unit cell, Z 4

Temperature 293(2)

Absorption coefficient 0.069 mm^-1^

F(000) 744

Scan mode ω scan

θ range for entire data collection 3.61 <θ< 25.00º

Range of indices h= -11 to 11, k= -26 to 26, l= -11 to 11

Reflections collected / unique 34328 / 3503

Reflections observed (I > 2σ(I)) 2672

R_int_  0.0377

R_sigma_  0.0210

Structure determination Direct methods

Refinement Full-matrix least-squares on F^2^

No. of parameters refined 333

Final R 0.0438

wR(F^2^) 0.1020

Weight 1/[σ ^2^(F_o_^2^)+( 0.0444P)^2^+0.5247P]

where P=[F_o_^2^ +2F_c_^2^] / 3

Goodness-of-fit 0.999

( Δ /σ )_max_ 0.001 ( x H8 )

Final residual electron density −0.120 < Δρ < 0.125 Å^−3^

Measurement *X’calibur system – Oxford diffraction make,*

*U.K.*

Software for structure solution: SHELXS97 ^[1]^

Software for refinement: SHELXL97 ^[2]^

Software for molecular plotting: ORTEP-3 [3]

PLATON [5]

Software for geometrical calculation PLATON [5]

PARST [4]

__________________________________________________________________

[1] Sheldrick GM (1997) SHELXS97, Program for the Solution of Crystal Structures, University of Gottingen, Gottingen, Germany

[2] Farrugia LJ (1997) ORTEP-3 for windows—A version of ORTEP-III with a graphical user interface (GUI). J Appl Cryst 30:565−566

### [3] Farrugia LJ (1999) **WinGX suite for small-molecule single-crystal crystallography**

J Appl Cryst 32:837−838

[4] Nardelli M (1995) PARST95-An update to PARST. A system of Fortran routines for calculating molecular structure parameters from the results of the crystal structure analysis. J Appl Cryst 28:659

### [5] Spek AL (2009) Structure validation in chemical crystallography Acta Cryst D65:148−155

*Copies of ^1^H- and ^13^C-NMR spectra of all the compounds including all entries are given below:*

*Bis*(*bis*-phenylmethyl)ether (1):

White solid, 86% yield, m.p. 106−107 ^o^C (Lit. 105−107 ^0^C [29]. 107 ^0^C [18]). IR (ν_max_, KBr) cm^−1^: 3057, 3028, 2953, 1595, 1489, 1445, 1250, 1163, 1098, 1072, 1029, 698. ^1^H NMR (CDCl_3_, 200 MHz, δ): 7.40-7.23 (m, 20H, Ar H), 5.41 (s, 2H, CH). ^13^C NMR (CDCl_3_, 100 MHz, δ): 142.28, 128.45, 127.51, 127.33, 80.05. TOF-MS: 373.44 ([M + Na]^+^). Anal. Found: C, 89.13; H, 6.28. C_26_H_22_O requires C, 89.11; H, 6.33%.

*
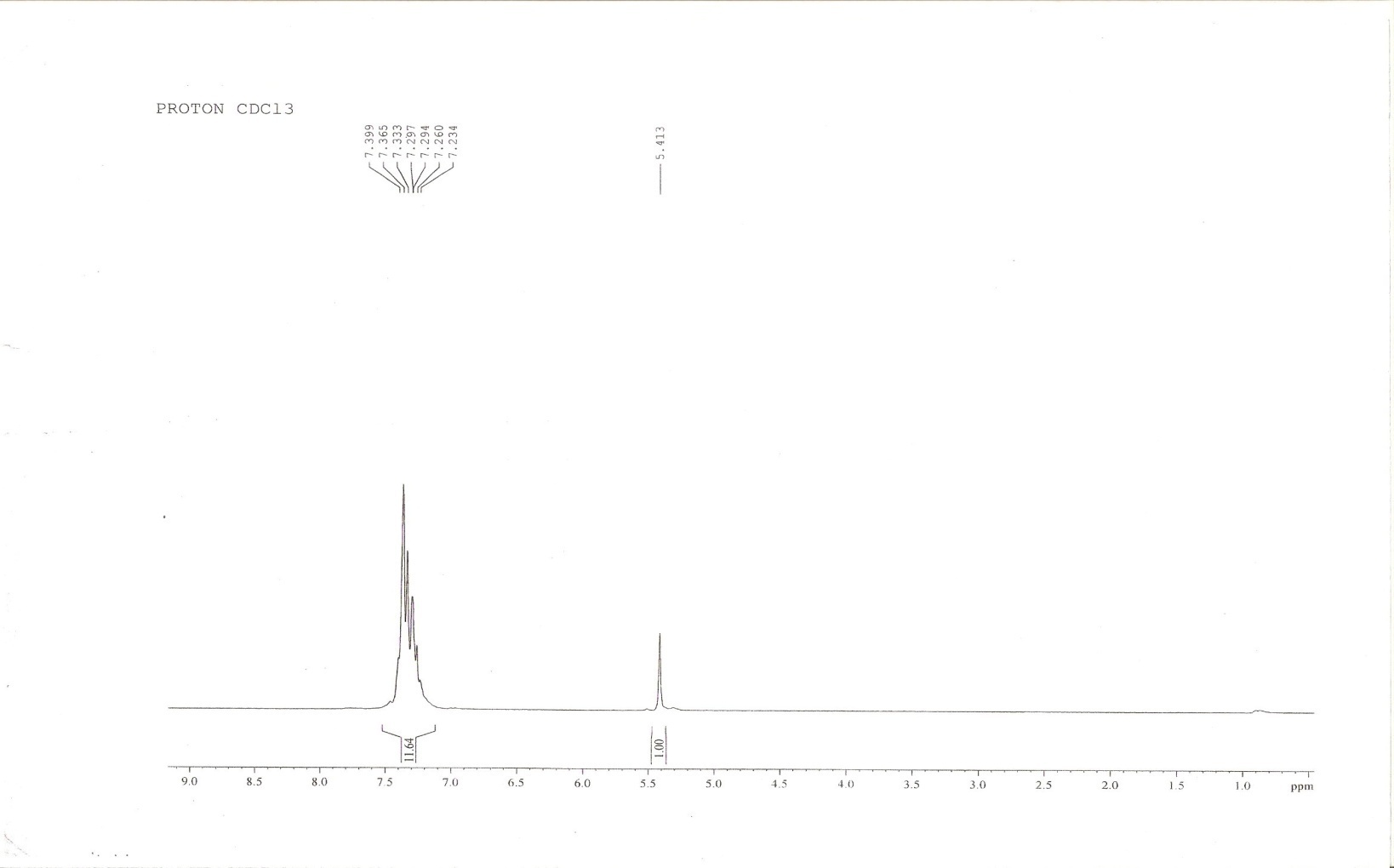
*

^1^H NMR spectrum of compound **1**

*
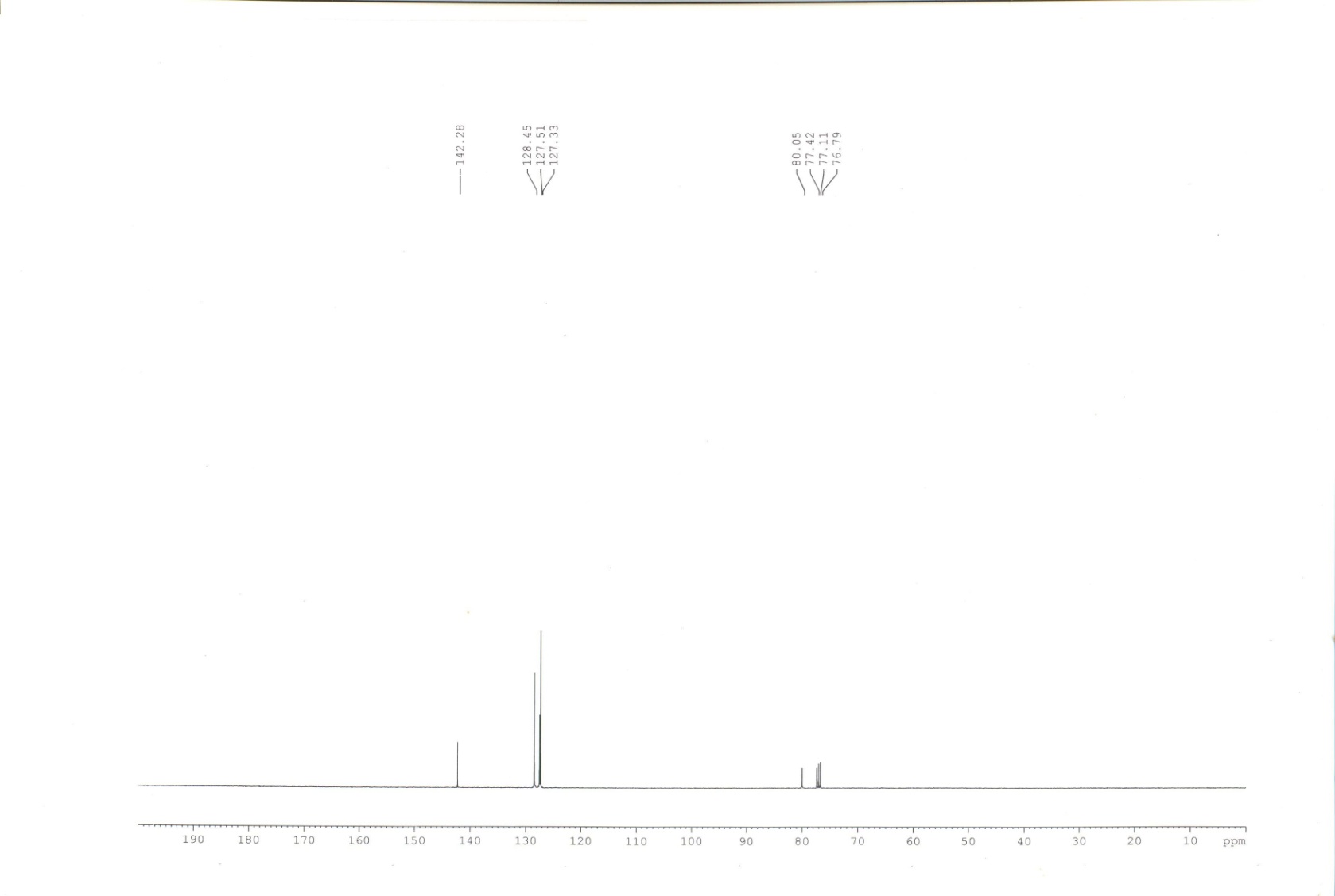
*

^13^C NMR spectrum of compound **1**

*Bis*[[1-(4-methylphenyl)-1-phenyl]methyl]ether (2):

Yellowish white, semi solid, 89% yield. IR (ν_max_, KBr) cm^−1^: 3060, 3025, 2923, 2852, 1655, 1460, 1277, 1124, 1071, 810, 824, 699. ^1^H NMR (CDCl_3_, 400 MHz, δ): 7.6 (d, 4H, Ar H, *J* = 7.6 Hz), 7.53-7.45 (m, 8H, Ar H), 7.43-7.41 (m, 2H, Ar H), 7.34 (d, 4H, Ar H, *J* = 7.6 Hz), 5.63 (s, 2H, CH), 2.53 (s, 6H, CH_3_). ^13^C NMR (CDCl_3_, 100 MHz, δ): 142.82, 142.70, 139.59, 139.48, 137.26, 137.22, 129.33, 129.30, 128.57, 128.54, 127.53, 127.49, 127.46, 127.41, 127.34, 79.96, 21.37. TOF-MS: 401.05 ([M + Na]^+^). Anal. Found: C, 89.89; H, 6.90. C_28_H_26_O requires C, 88.85; H, 6.92%.


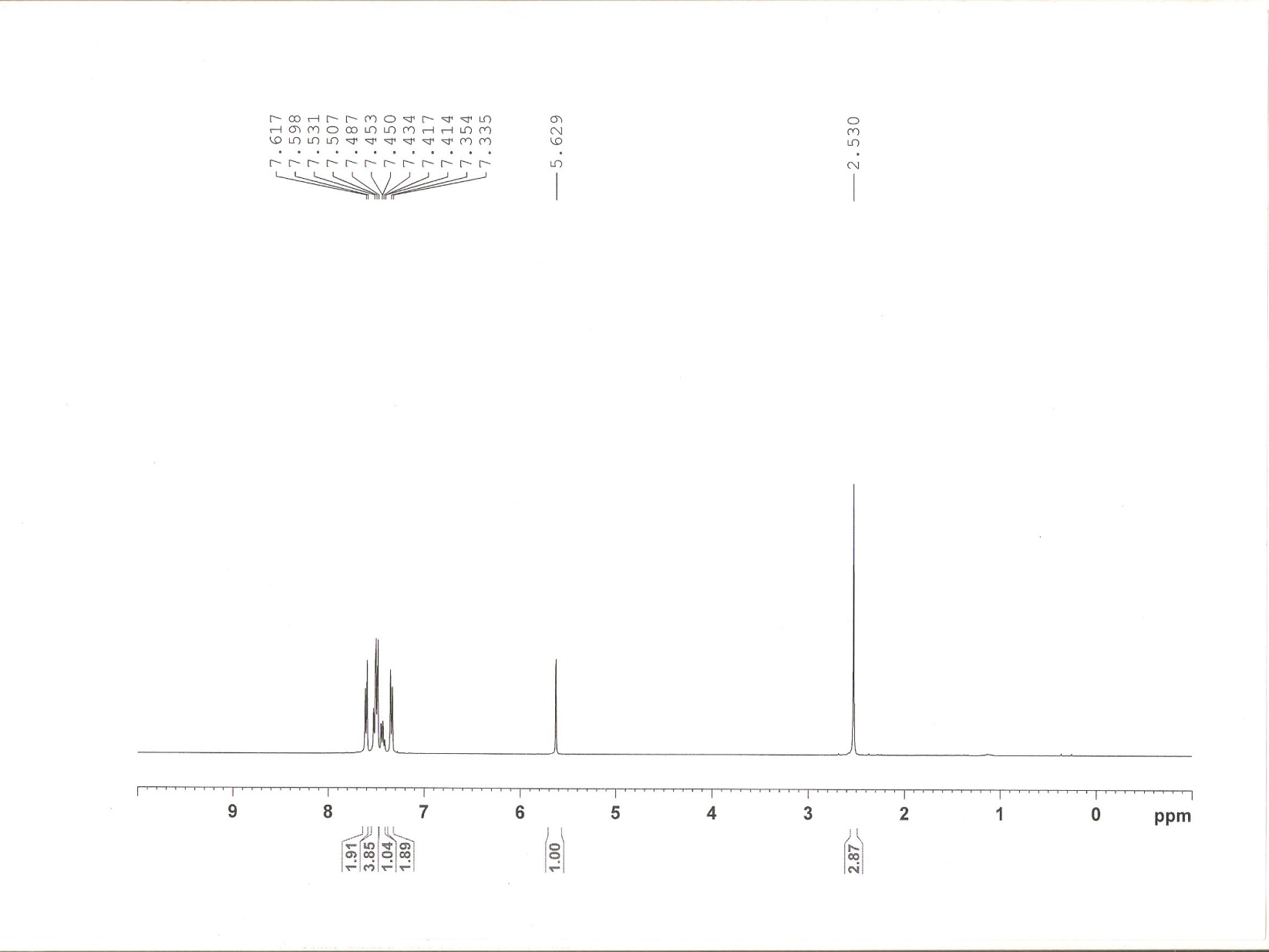


^1^H NMR spectrum of compound **2**


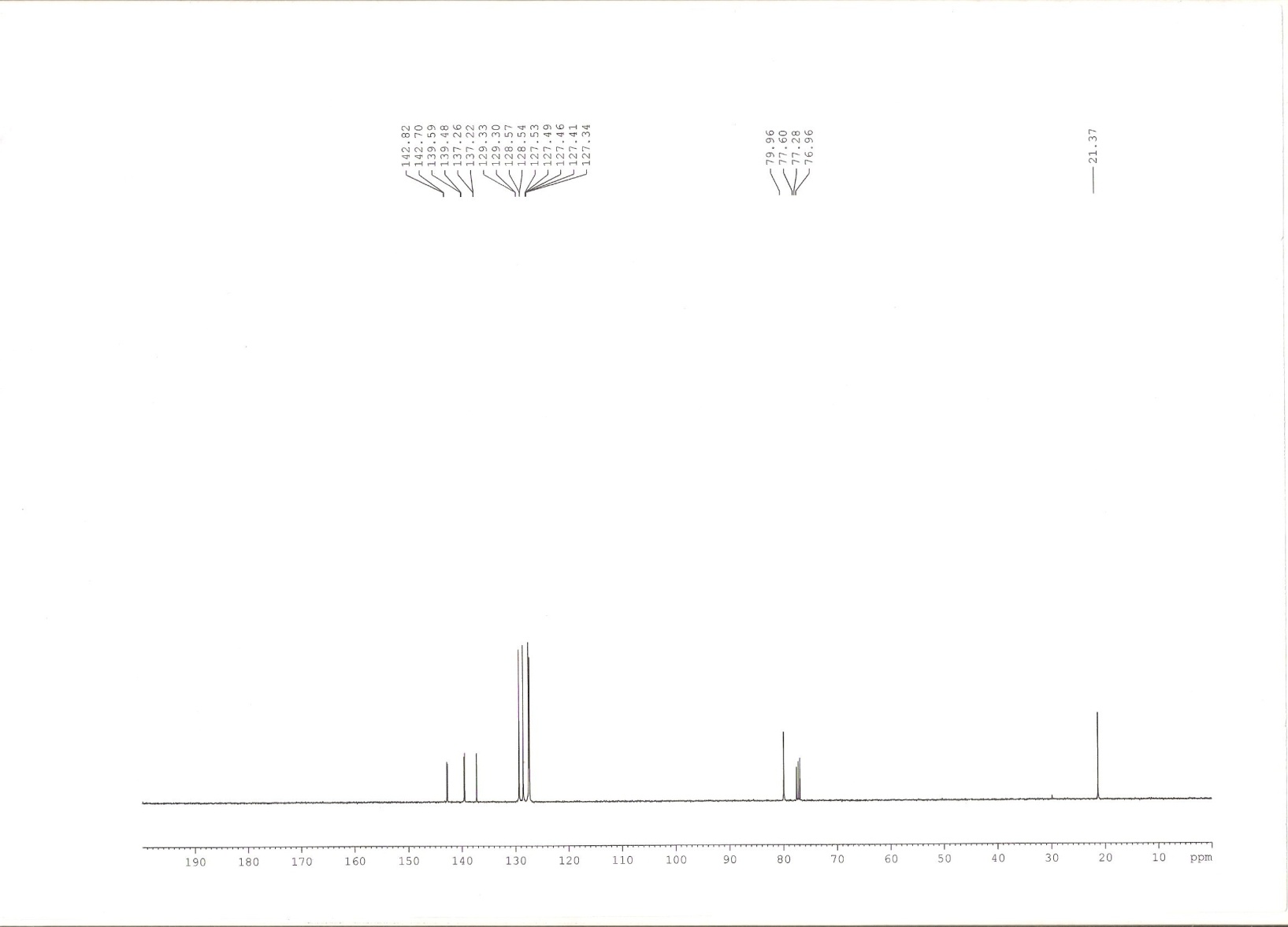


^13^C NMR spectrum of compound **2**

*Bis*[[1-(4-chlorophenyl)-1-phenyl]methyl]ether (3):

White semi solid, 85% yield. IR (ν_max_, KBr) cm^−1^: 3063, 3029, 2925, 2854, 1595, 1490, 1449, 1259, 1185, 1086, 1057, 843, 811, 700. ^1^H NMR (CDCl_3_, 400 MHz, δ): 7.31-7.30 (m, 8H, Ar H), 7.28-7.25 (m, 10H, Ar H), 5.33 (s, 2H, CH). ^13^C NMR (CDCl_3_, 100 MHz, δ): 141.49, 141.39, 140.65, 140.54, 133.37, 133.30, 128.69, 128.63, 128.60, 128.56, 128.48, 127.87, 127.81, 127.22, 127.13, 79.53. TOF-MS: 441.94 ([M + Na]^+^). Anal. Found: C, 74.45; H, 4.83. C_26_H_20_Cl_2_O requires C, 74.47; H, 4.81%.


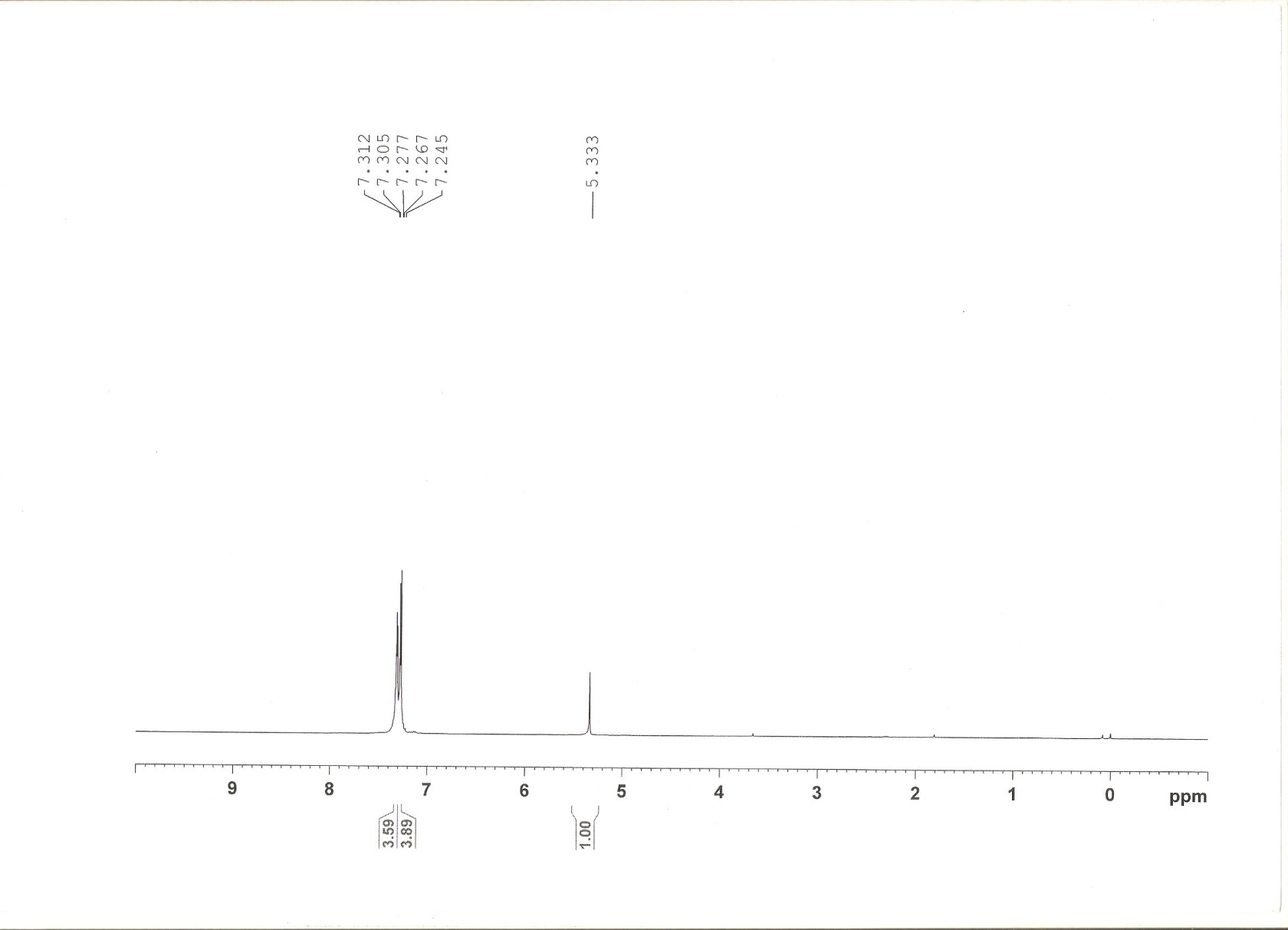


^1^H NMR spectrum of compound **3**

*
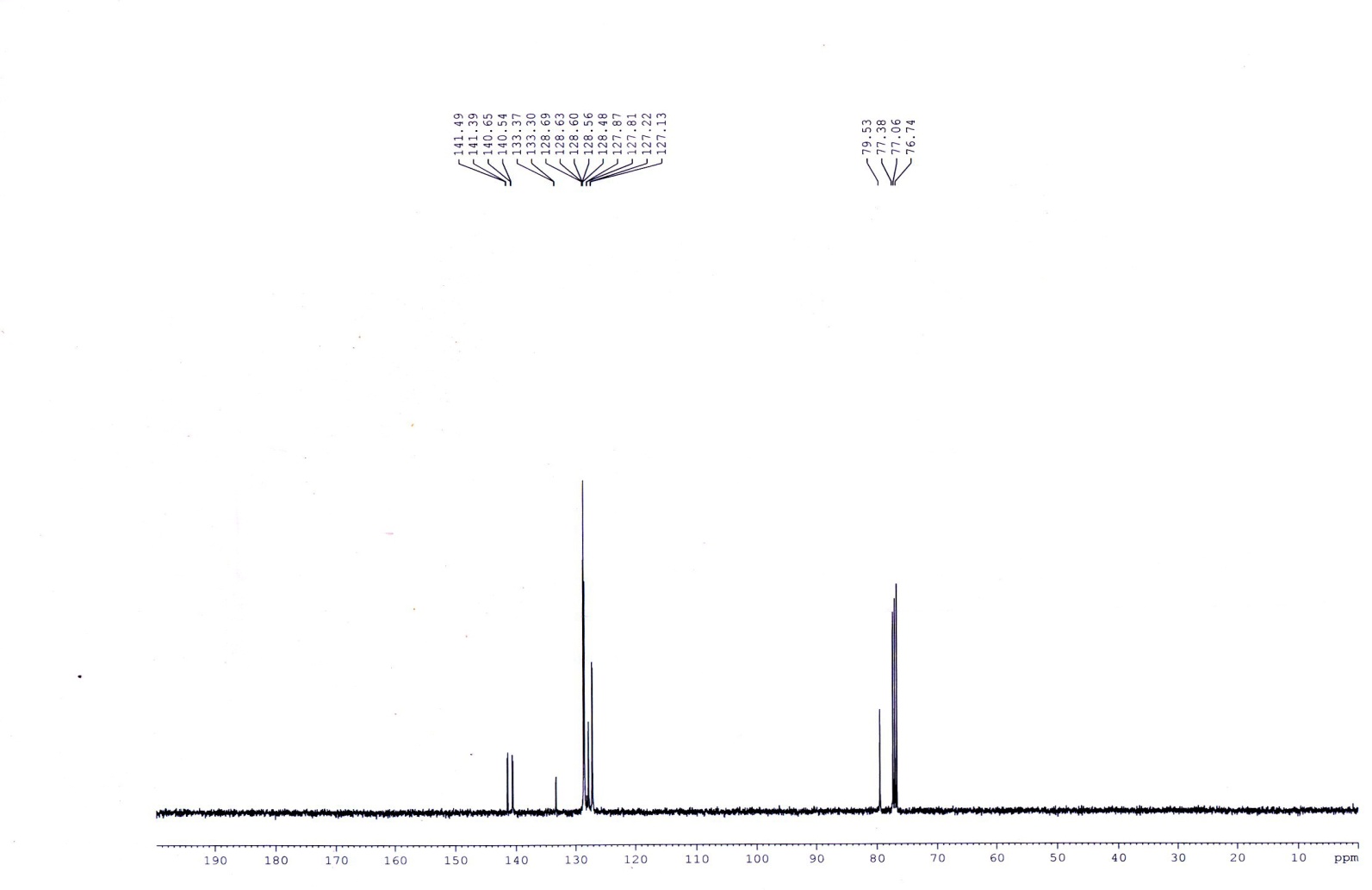
*

^13^C NMR spectrum of compound **3**

*Bis*[[1-(4-bromophenyl)-1-phenyl]methyl]ether (4):

White semi solid, 92% yield. IR (ν_max_, KBr) cm^−1^: 3085, 3062, 3028, 2924, 2854, 1602, 1590, 1486, 1454, 1290, 1185, 1107, 1070, 1028, 847, 793, 700. ^1^H NMR (CDCl_3_, 400 MHz, δ): 7.33 (dd, 4H, Ar H, *J* = 8.4, 5.2 Hz), 7.21-7.15 (m, 10H, Ar H), 7.12 (dd, 4H, Ar H, *J* = 8.4, 3.2 Hz), 5.23 (s, 2H, CH). ^13^C NMR (CDCl_3_, 100 MHz, δ): 141.43, 141.33, 141.20, 141.08, 131.68, 131.62, 128.94, 128.86, 128.70, 128.65, 127.94, 127.87, 127.26, 127.17, 121.60, 121.52, 79.61. TOF-MS: 528.74 ([M + Na]^+^). Anal. Found: C, 61.49; H, 3.93. C_26_H_18_Br_2_O requires C, 61.44; H, 3.97%.

*
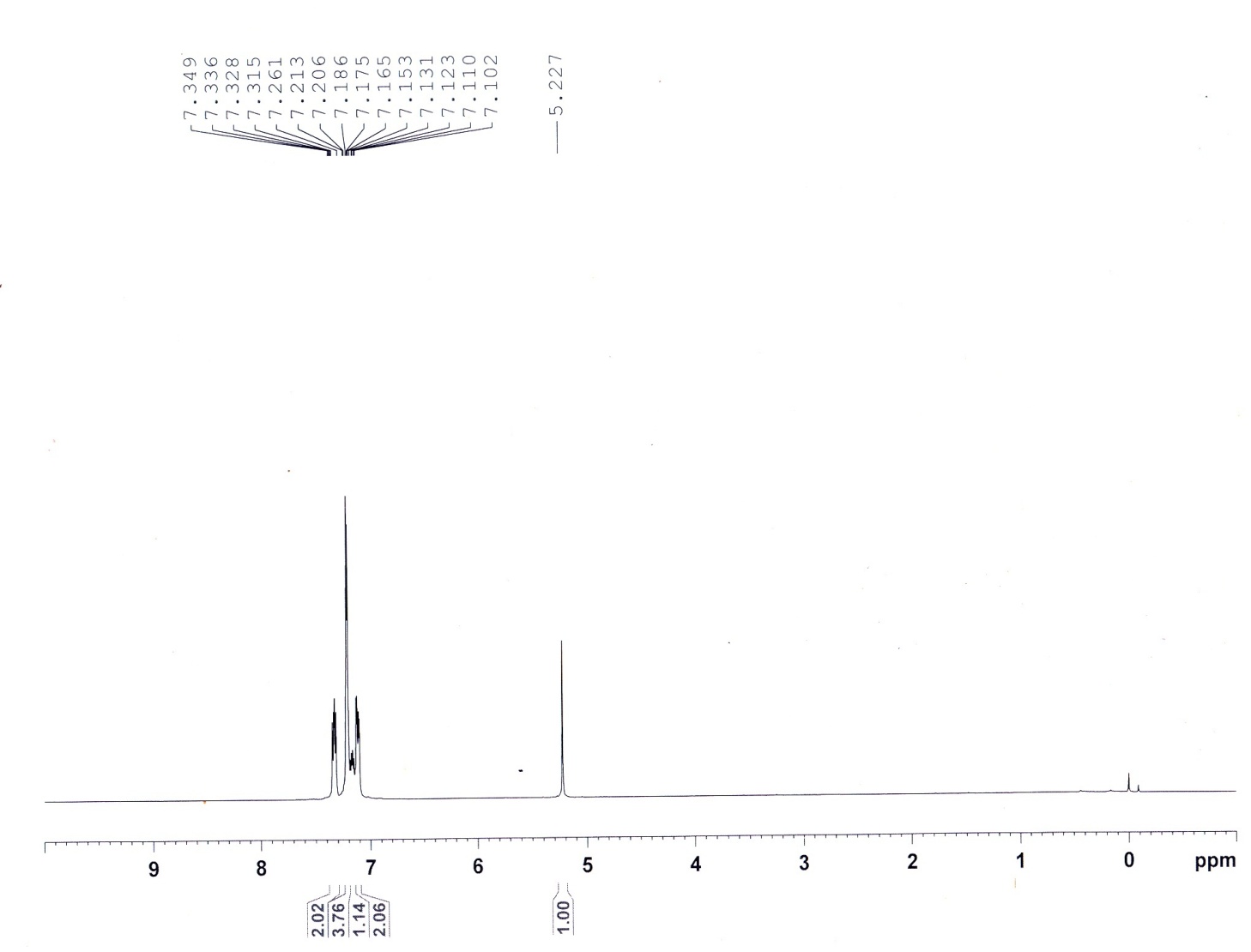
*

^1^H NMR spectrum of compound **4**

*
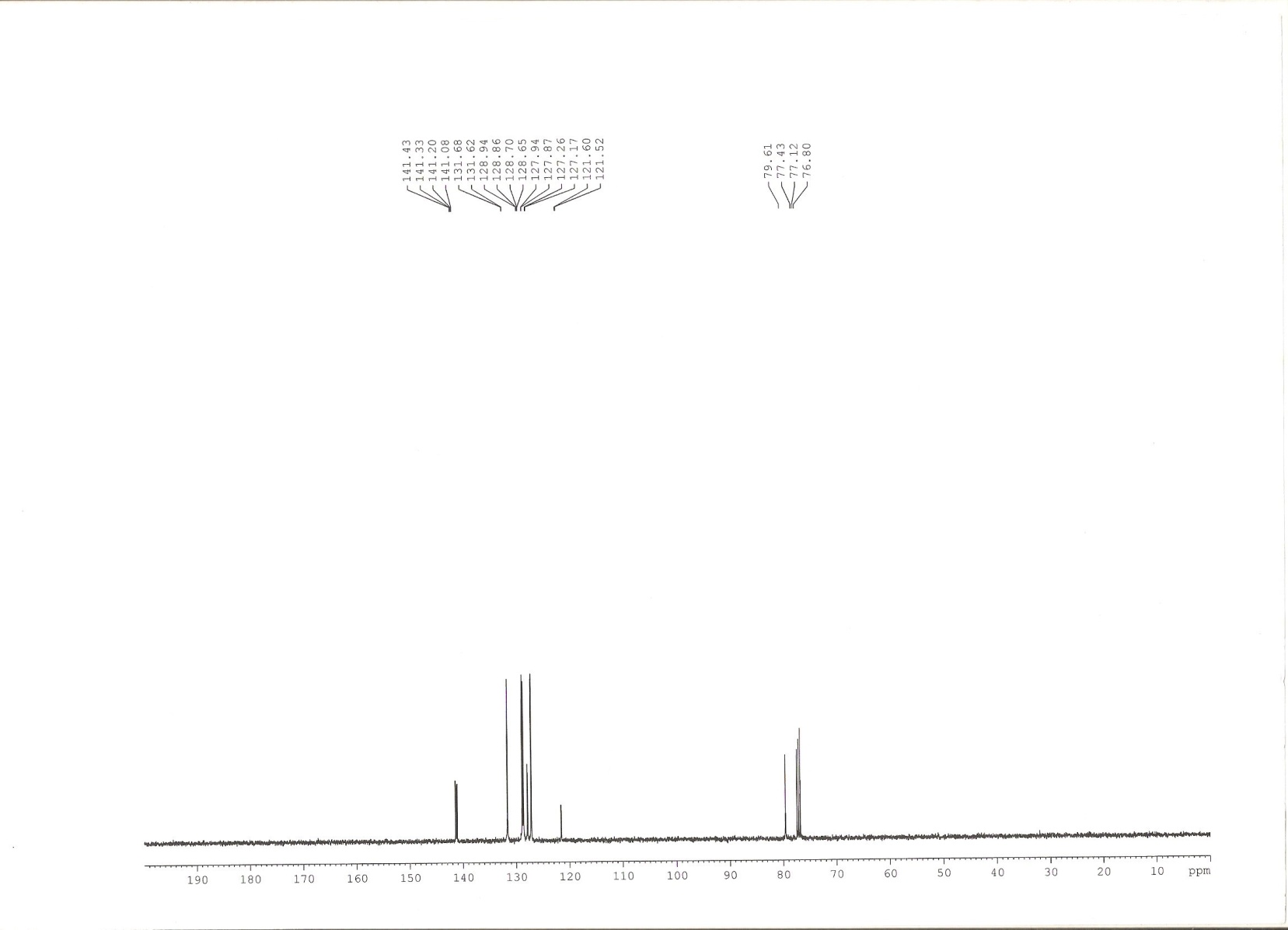
*

^13^C NMR spectrum of compound **4**

*Bis*[*bi*s(4-chlorophenyl)methyl]ether (5):

White solid, 88% yield, m.p. 125-127 ^o^C (Lit. 126-127 ^o^C) [30, 31]. IR (ν_max_, KBr) cm^−1^: 3031, 2924, 1594, 1491, 1410, 1290, 1188, 1089, 1013, 854, 824, 735, 726. ^1^H NMR (CDCl_3_, 200 MHz, δ): 7.31 (d, 8H, Ar H, *J* = 8.6 Hz), 7.23 (d, 8H, Ar H, *J* = 8.6 Hz), 5.29 (s, 2H, CH). ^13^C NMR (CDCl_3_, 75 MHz, δ): 139.72, 133.71, 128.82, 128.36, 78.97. TOF-MS: 509.12 ([M + Na]^+^). Anal. Found: C, 63.94; H, 3.69; C_26_H_18_Cl_4_O requires C, 63.96; H, 3.72%.


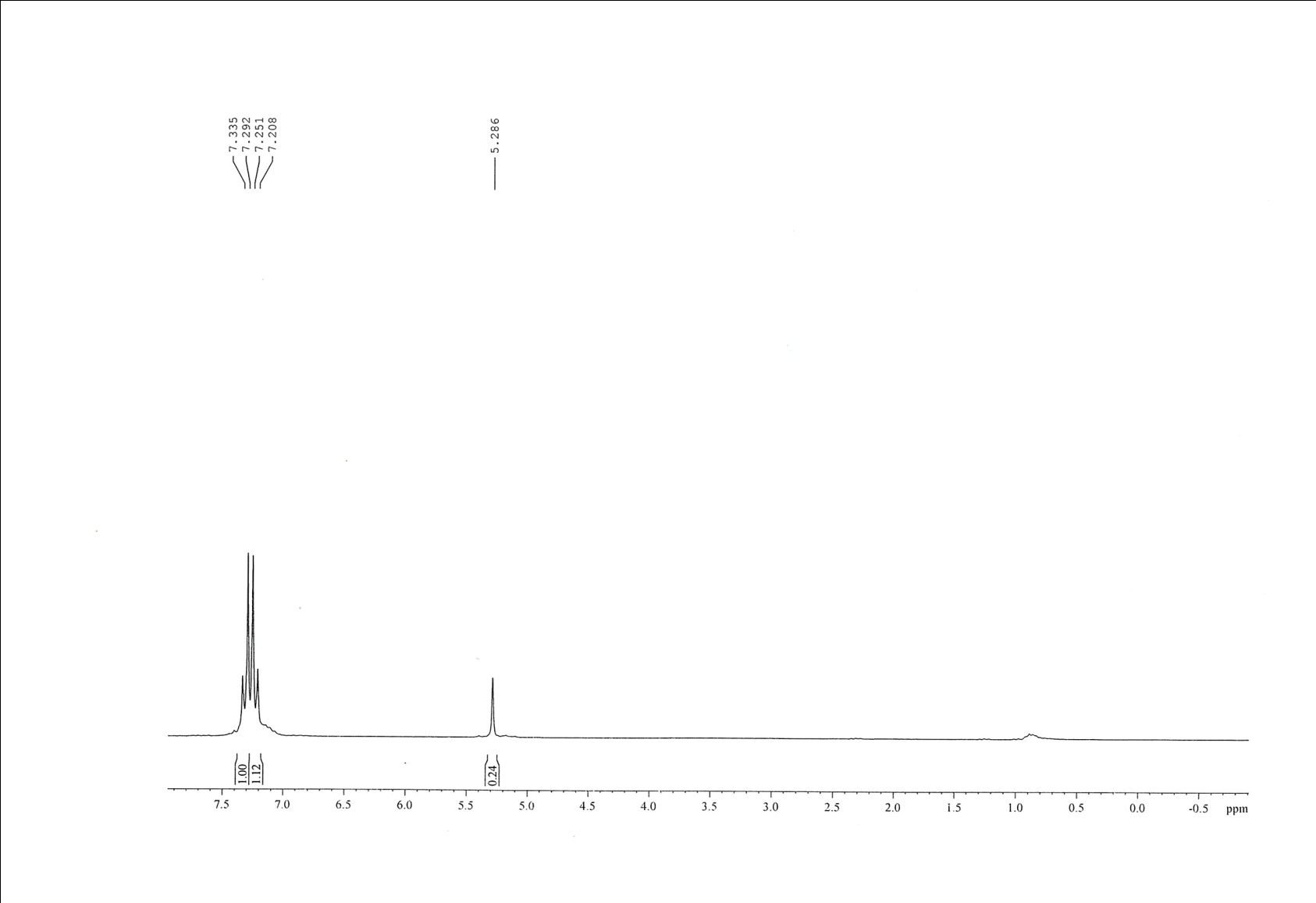


^1^H NMR spectrum of compound **5**


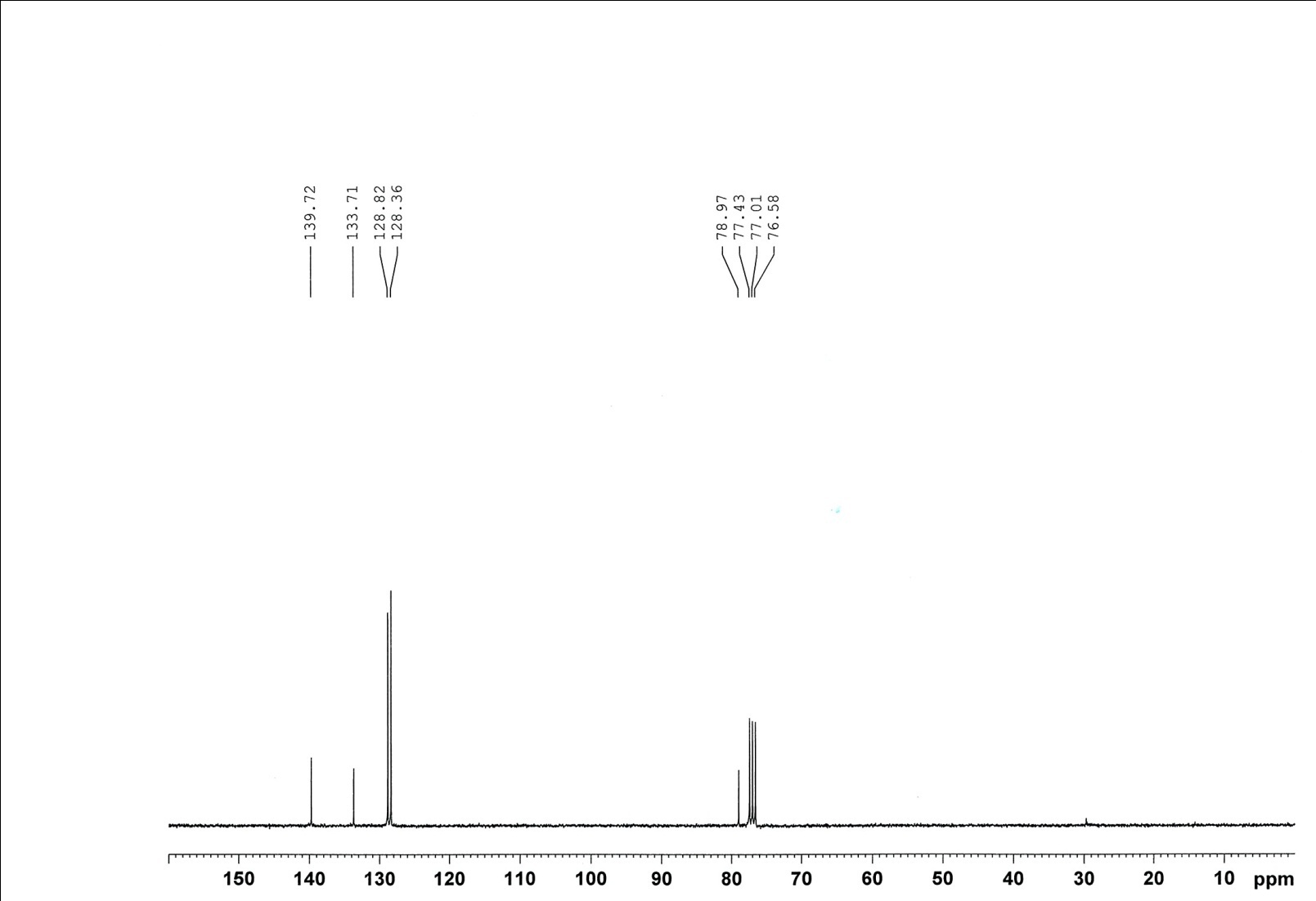


^13^C NMR spectrum of compound **5**

*Bis*[*bis*[4-fluorophenyl]methyl]ether (6):

White solid, 91% yield, m.p. 88-90 ^o^C. IR (ν_max_, KBr) cm^−1^: 3069, 3057, 2925, 1603, 1507, 1422, 1408, 1298, 1225, 1178, 1155, 1101, 1029, 859, 837, 818. ^1^H NMR (CDCl_3_, 400 MHz, δ): 7.19-7.16 (m, 8H, Ar H), 6.94-6.88 (m, 8H, Ar H), 5.22 (s, 2H, CH). ^13^C NMR (CDCl_3_, 100 MHz, δ): 163.52, 161.07, 137.51, 137.48, 128.82, 128.74, 115.59, 115.38, 78.91. TOF-MS: 445.98 ([M + Na]^+^). Anal. Found: C, 73.89; H, 4.28. C_26_H_18_F_4_O requires C, 73.93; H, 4.30%.


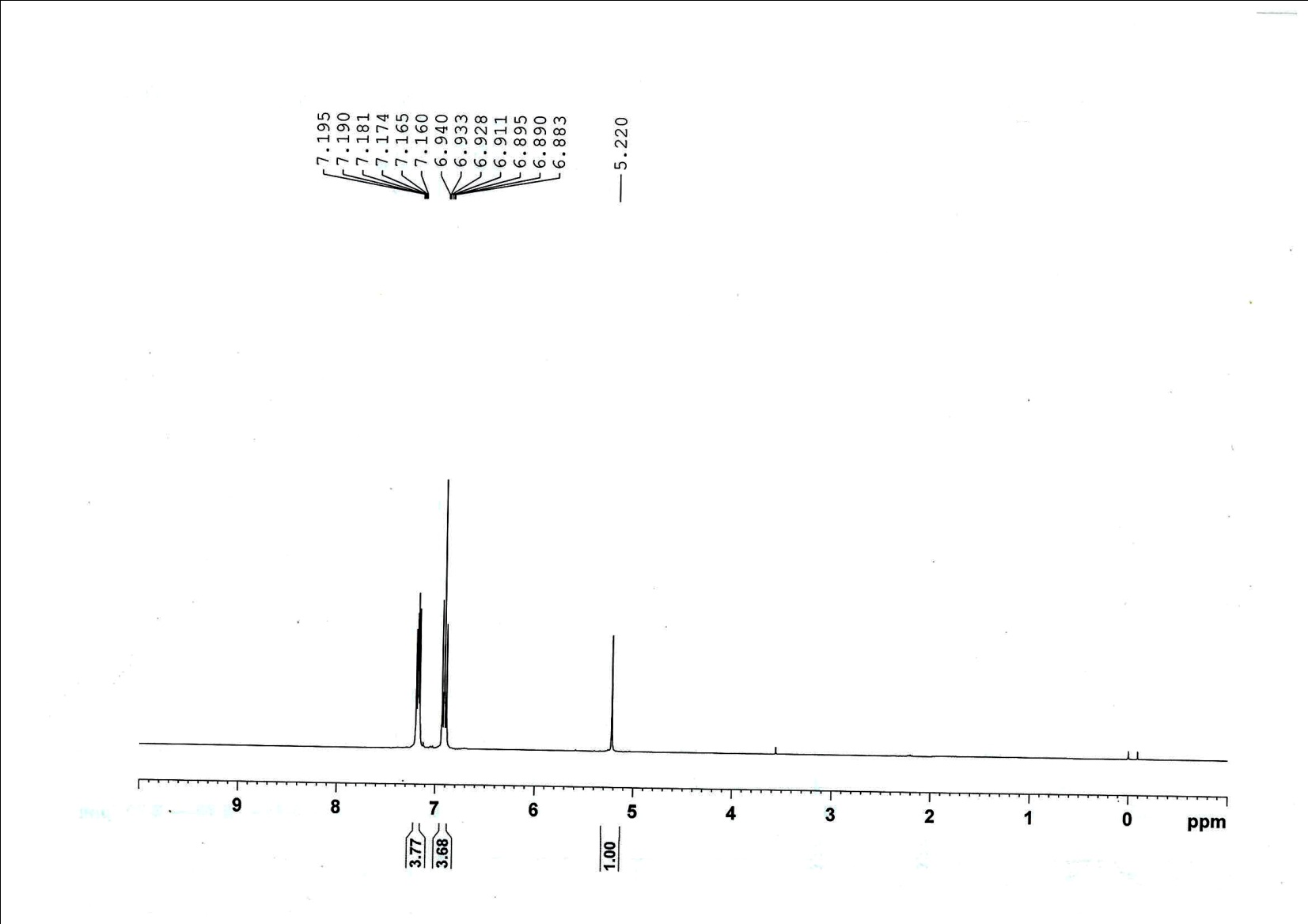


^1^H NMR spectrum of compound **6**

*
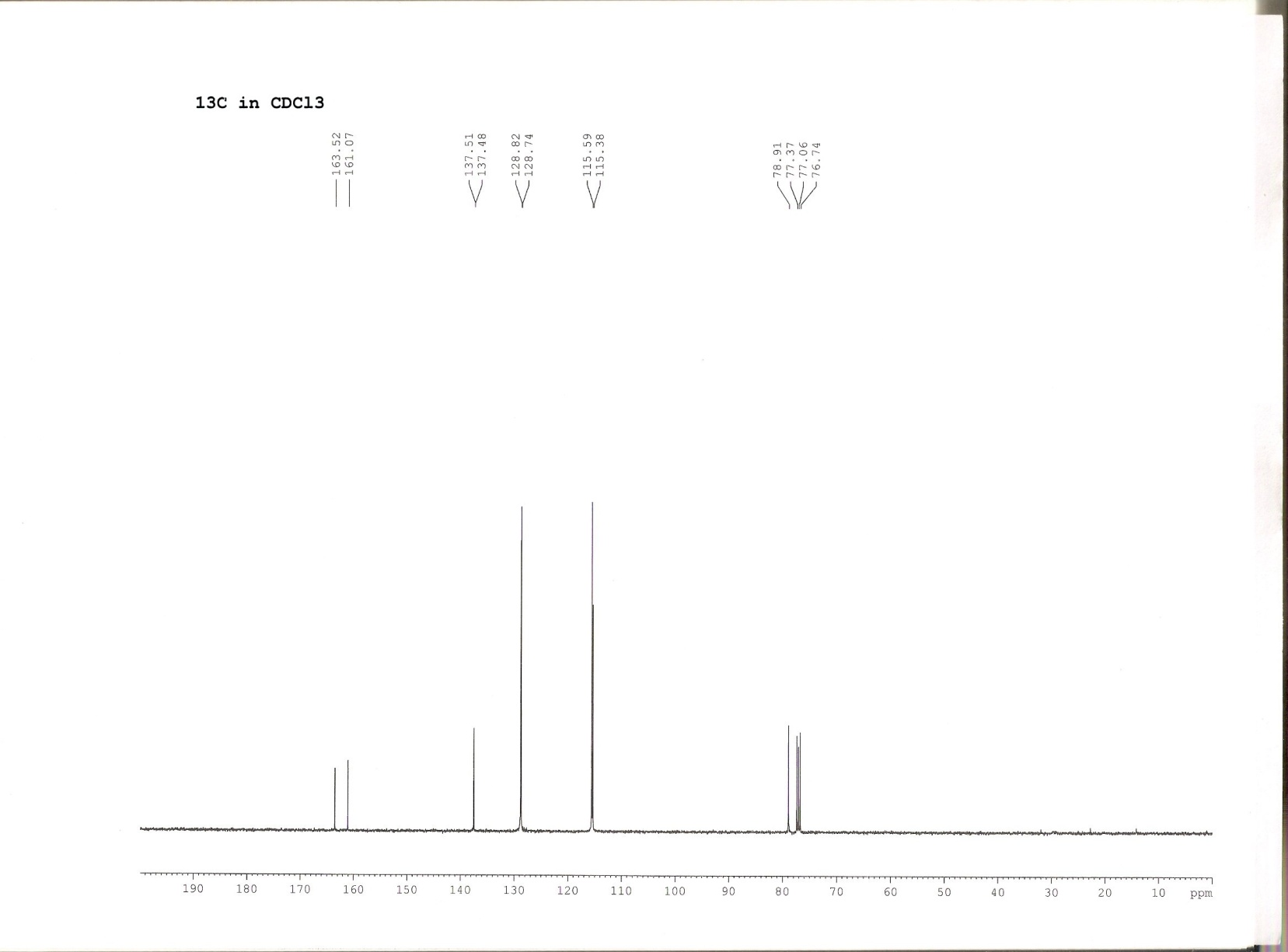
*

^13^C NMR spectrum of compound **6**

*Bis*[[1-(4-methoxyphenyl)-1-phenyl]methyl]ether (7):

Colorless liquid, 90% yield. IR (ν_max_, KBr) cm^−1^: 3062, 3029, 2953, 2932, 2906, 2835, 1510, 1510, 1494, 1451, 1249, 1171, 1111, 1080, 849, 819, 698. ^1^H NMR (CDCl_3_, 400 MHz, δ): 7.35 (d, 4H, Ar H, *J* = 7.6 Hz), 7.32-7.28 (m, 4H, Ar H), 7.27-7.24 (m, 6H, Ar H), 6.84 (d, 4H, Ar H, *J* = 8.4 Hz), 5.34 (s, 2H, CH), 3.77 (s, 6H, OCH_3_). ^13^C NMR (CDCl_3_, 100 MHz, δ): 158.97, 158.93, 142.72, 142.52, 134.53, 134.32, 128.67, 128.60, 128.35, 128.32, 127.30, 127.24, 127.17, 127.09, 113.80, 113.77, 79.43, 79.40, 55.26. TOF-MS: 432.99 ([M + Na]^+^). Anal. Found: C, 81.95; H, 6.37. C_28_H_26_O_3_ requires C, 81.92; H, 6.38%.

**
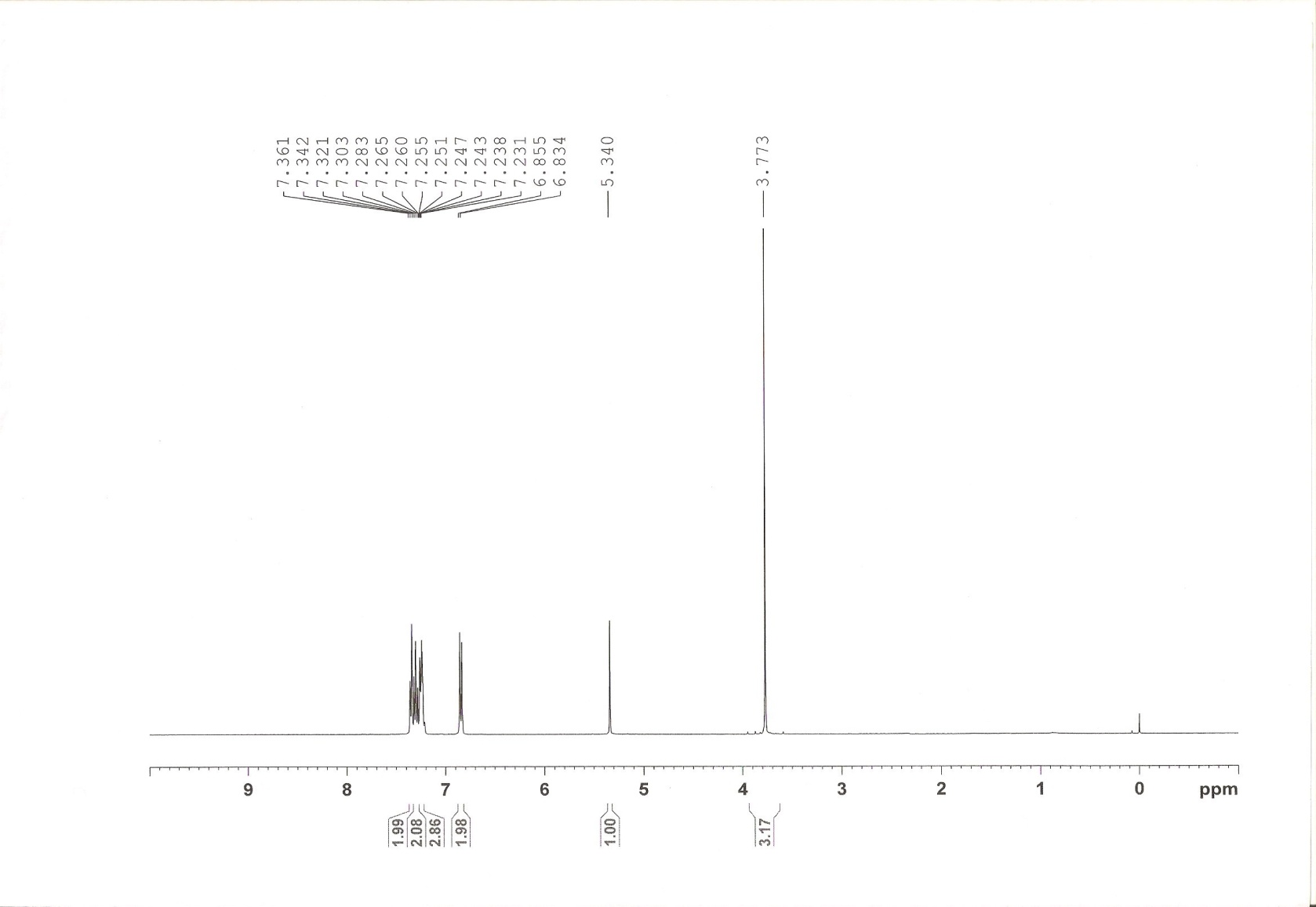
**

^1^H NMR spectrum of compound **7**


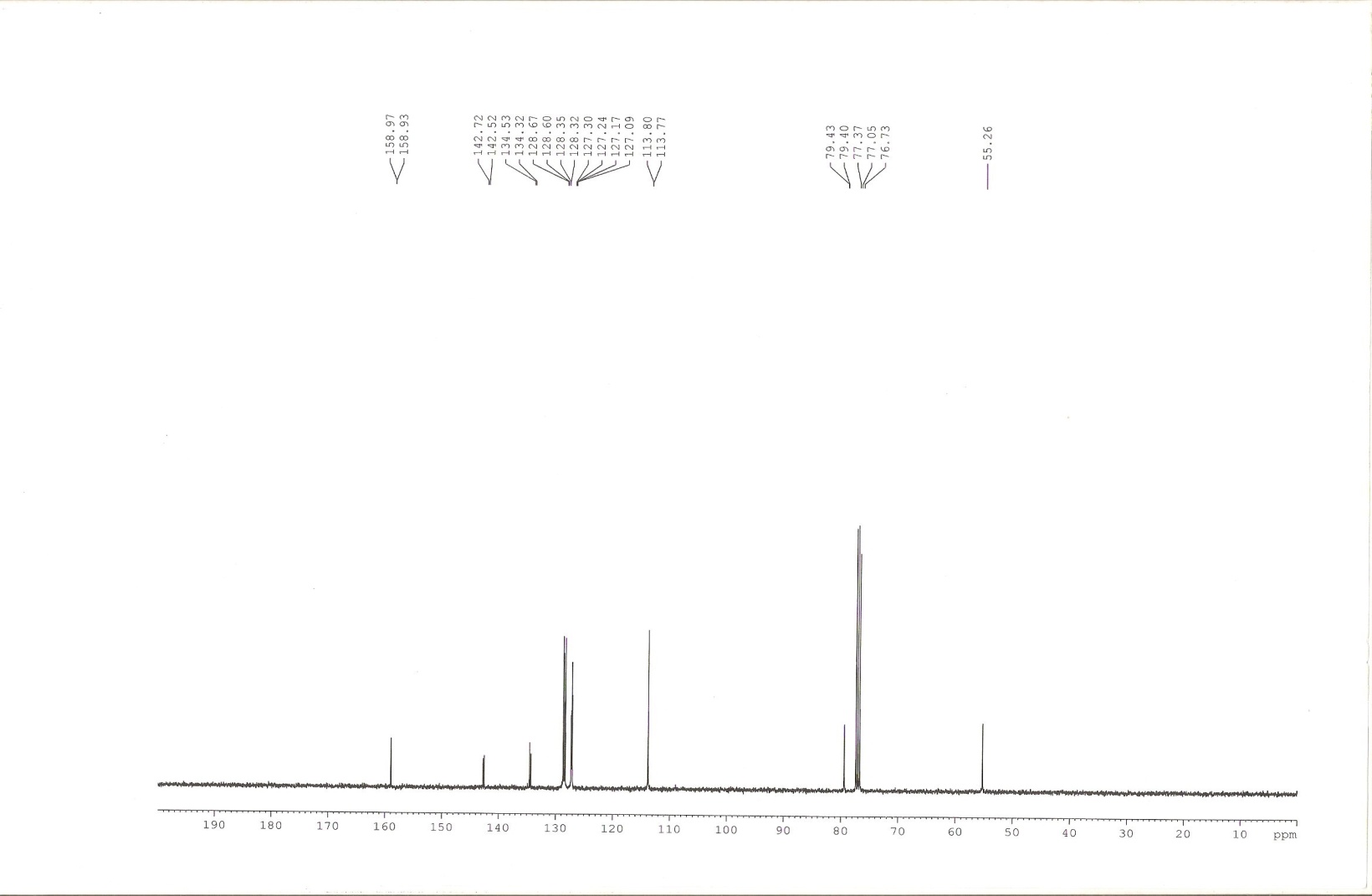


^13^C NMR spectrum of compound **7**

___________
